# Glutamine protects against oxidative stress injury through inhibiting the activation of PI3K/Akt signaling pathway in parkinsonian cell model

**DOI:** 10.1186/s12199-018-0757-5

**Published:** 2019-01-05

**Authors:** Yingqian Zhao, Qiang Wang, Yuan Wang, Jie Li, Gang Lu, Zhibin Liu

**Affiliations:** 1 Nanjing University of Chinese Medicine, 138 Xianlin Avenue, Nanjing, China; 2 College of Acupuncture and Moxibustion, Shaanxi University of Chinese Medicine, Qindu District, Xianyang, China; 3 Innovation Research Center of Acupuncture and Medicine, Shaanxi University of Chinese Medicine, Qindu District, Xianyang, Shaanxi China; 4 Shaanxi Key Laboratory of Acupuncture and Medicine, Qindu District, Xianyang, Shaanxi China

**Keywords:** Parkinson’s disease, Oxidative stress, Glutamine, PC12, PI3K/Akt

## Abstract

**Background:**

Parkinson’s disease is a neurodegenerative disorder, and recent studies suggested that oxidative stress contributes to the degeneration of dopamine cell in Parkinson’s disease. Glutamine also has a positive role in reducing oxidative stress damage. In this study, we hypothesized that glutamine offers protection against oxidative stress injury in 1-methyl-4-phenylpyridinium (MPP^+^)-induced Parkinson’s disease cell model.

**Methods:**

MPP^+^ was used to induce PD models in PC12 cells and classified into control, M0 (MPP^+^), G0 (glutamine), and M0+G0 groups. CCK-8 and AO/EB staining assays were used to examine cell proliferation and apoptosis, respectively. Western blotting was applied to examine the protein expression of PI3K, P-Akt, Akt, P-mTOR, and mTOR.

**Results:**

We showed that glutamine suppressed cytotoxicity induced by MPP^+^ in PC12 cells. MPP^+^ decreased the superoxide dismutase and glutathione peroxidase activity and increased the malondialdehyde content, which were restored by glutamine. Moreover, MPP^+^ increased the expression of PI3K, P-Akt, Akt, P-mTOR, and mTOR, which were inhibited by glutamine. And the antioxidant capacity of glutamine on PC12 cells could be improved by LY294002 and inhibited by IGF-1.

**Conclusion:**

These results suggest that glutamine strengthens the antioxidant capacity in PC12 cells induced by MPP^+^ through inhibiting the activation of the PI3K/Akt signaling pathway. The effects of glutamine should be investigated and the protective mechanism of glutamine in PD must be explored in future studies.

## Introduction

Parkinson’s disease (PD) is a neurodegenerative disorder caused by the progressive loss of dopaminergic neurons, with a considerably high incidence and mortality among people over the age of 65 [1, 2]. According to the latest Movement Disorder Society (MDS) PD Criteria, bradykinesia is a core motion symptom of PD patients, accompanied by static tremor and/or muscular rigidity [3]; the non-motor symptoms of PD including sleep disturbance, smell disturbance, and mental disturbance [4]. The key pathological changes of PD include two aspects: one is the degeneration and loss of dopaminergic neurons caused by environmental factors and various molecular pathways, including oxidative stress, mitochondrial dysfunction, inflammatory, or immune response; the other aspect is the formation of lewy body [5]. Oxidative stress, regarded as an imbalance in the production of reactive oxygen species and the ability of the cell to elicit an effective antioxidant response [6], has demonstrated it is closely related to Alzheimer’s disease and amyotrophic lateral sclerosis [7, 8]. Similarly, in many PD cases, oxidative stress seems to be a potential mechanism that induced cell dysfunction and death [9].

Glutamine (Gln) is the most abundant free amino acid in the human body, which has extremely fast cell turnover rate and extensive physiological functions [10]. Gln is the main energy supply substance for mitochondria to form ATP; also, the oxidation of Gln can eliminate some strong oxidizing substances in cells and protect some important components of cells from oxidative damage [11]. At present, studies have shown that supplementing exogenous Gln is able to meet the needs of the human body, maintain the stability of the internal environment, and prevent and reduce multiple organ dysfunction syndromes [12]. In clinical practice, some scholars have increased glutamine content in the diet of patients with Alzheimer’s disease, Huntington’s disease, and amyotrophic lateral sclerosis, so as to reduce oxidative stress damage [13, 14]. However, the role of glutamine in PD is not clear.

The phosphatidylinositol-3-kinase (PI3K)/protein kinase B (Akt)/mammalian target of rapamycin (mTOR) pathway plays a vital regulatory role in the occurrence and development of oxidative stress, and the role of oxidative stress in Parkinson’s disease is related to this pathway [15, 16]. Thus, our study aimed to evaluate whether glutamine protects against oxidative stress-induced injury via activation of the PI3K/Akt/mTOR signaling pathway in a cell model of PD.

## Materials and methods

### Reagents

1-Methyl-4-phenylpyridinium (MPP^+^) was purchased from Sigma-Aldrich (Merck KGaA, Darmstadt, Germany) and the purity of MPP^+^ was ≥ 97%. LY294002 (PI3K/Akt inhibitor) and glutamine (Gln) also were obtained from Sigma-Aldrich, and the LY294002 was used at a concentration of 20 μM. IGF-1 was purchased from Apexbio Technology LLC (Houston, TX, USA) and used at a concentration of 100 ng/ml. Additional reagents employed in the present study were commercially available and of analytical purity.

### Cell culture

Cells of the rat pheochromocytoma tumor cell line PC12 were purchased from Procell Life Science & Technology Co., Ltd. (Wuhan, China). PC12 cells were cultured in RPMI-1640 medium (without glutamine; Procell Life Science & Technology Co., Ltd., Wuhan, China) supplemented with 10% fetal bovine serum (Clark Bioscience, MD, USA), 5% horse serum (Hyclone, UT, USA), and 1% penicillin–streptomycin (Hyclone, UT, USA) at 37 °C in a humidified atmosphere containing 5% CO_2_.

### Cell viability by CCK-8 assay

PC12 cells were suspended and cultured in 96-well plates at a density of 6 × 10^3^/well. Then, cells were cultured in 10% Cell Counting Kit-8 (CCK-8; Dojindo Laboratories, Kumamoto, Japan) diluted in fresh medium for 1 h. The absorbance value was calculated by using a microplate reader (Thermo Fisher Scientific, MA, USA).

### AO/EB staining assay

PC12 cells were suspended and seeded onto chamber slides in six-well plates at a density of 2 × 10^5^/well and incubated for 48 h. Following treatment, the cells were washed with phosphate buffered saline three times and fixed in 4% paraformaldehyde for 20 min. Cell nuclei were stained with acridineorange (AO) and ethidium bromide (EB) for 5 min. The images of the cells were captured under a fluorescence microscope (Olympus Corporation, Tokyo, Japan).

### Western blot assay

The harvested cells were washed with phosphate buffered saline and lysed with lysis buffer (Boster, Wuhan, China) to obtain total cellular protein. Protein concentration was determined by using BCA Protein Assay kit (Boster, Wuhan, China). The protein samples were separated by 10% sodium dodecyl sulfate-polyacrylamide gel electrophoresis (SDS-PAGE) and transferred to a polyvinylidene fluoride (PVDF; Millipore Corporation, MA, USA) membrane through a Bio-Rad II System (Bio-Rad Laboratories, Inc.). Then, the membranes were sealed with 5% skimmed milk powder at room temperature for 1 h and incubated with rabbit monoclonal antibody against PI3K, P-Akt, Akt, P-mTOR, mTOR, and β-actin (1:1000; Cell Signaling Technology, MA, USA) at 4 °C overnight and goat anti-rabbit IgG at room temperature for 1 h. β-actin was used as inner loading control. The epitope was visualized by an ECL detection reagent (Millipore Corporation, MA, USA) according to the manufacturer’s instructions. The gray value was analyzed by Image-ProPlus software (Media Cybernetics, Inc., MD, USA).

### Detection of superoxide dismutase (SOD) and glutathione peroxidase (GSH-Px) activity and malondialdehyde (MDA) content

The harvested cells were washed with phosphate buffered saline twice and digested with trypsin. Following the cells was disrupted by an ultrasonic cell disruptor at 4 °C and the lysate was centrifuged at 1000 r/min at 4 °C for 10 min. A total of 100 μl supernatant was obtained to detect the OD values using a microplate reader according to the instructions of SOD, GSH-Px, and MDA kit, and the activity and content were calculated, respectively.

### Statistical analysis

Statistical evaluation was conducted using SPSS 20.0 (SPSS, Inc., Chicago, IL, USA). Values were presented as the means ± standard deviation. Differences among multiple groups were compared by one-way analysis of variance (ANOVA) with Dunnett’s post-tests or two-way ANOVA with Bonferroni’s post-tests. *p* < 0.05 was considered statistically significant.

## Results

### The cytotoxicity of MPP^+^ on PC12 cells

**Fig. 1:**
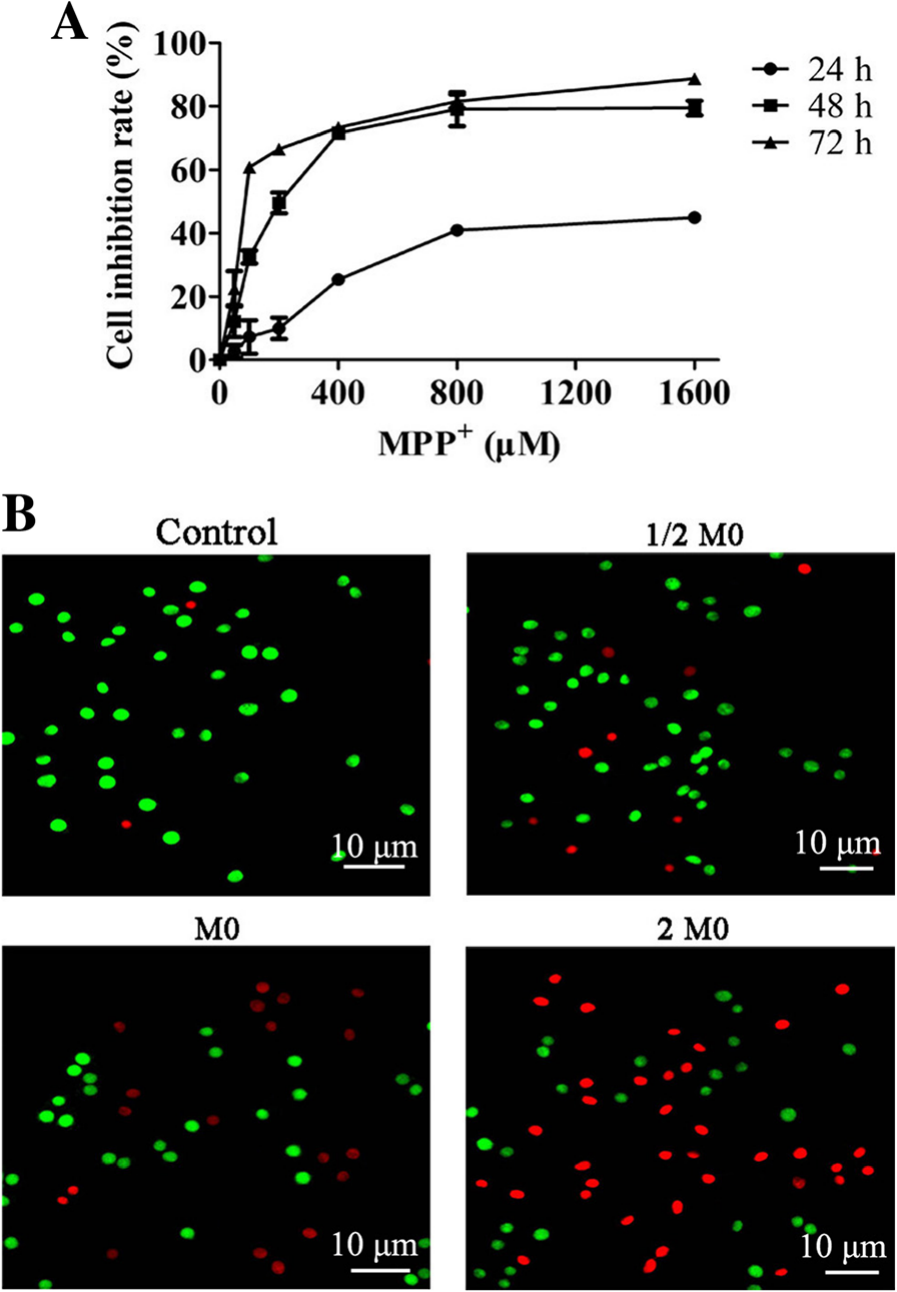
Effects of MPP^+^ on the proliferation and apoptosis of PC12 cells. **a** PC12 cells were incubated with MPP^+^ (0, 50, 100, 200, 400, 800, 1600 μM) for 24, 48, and 72 h. Cell viability was determined by a Cell Counting Kit-8 assay. Data were obtained from three independent experiments. The results were presented as the mean ± standard deviation. **b** Images of AO/EB staining were observed via fluorescence microscopy after treatment with MPP^+^ (113.5, 227, and 454 μM) for 48 h (× 400); the 113.5, 227, and 454 μM are showed as 1/2 M0, M0, and 2 M0, respectively

In order to study the cytotoxicity of MPP^+^ on PC12 cells, the cells were treated with various concentrations of MPP^+^ for 24, 48, and 72 h and the inhibition of cell proliferation was detected via a CCK-8 assay. As shown in Fig. 1a, MPP^+^ decreased the viability of PC12 cells in a dose- and time-dependent manner, with IC_50_ values of 1552.21 ± 125.38, 227.91 ± 23.08, and 109.82 ± 13.02 μM at 24, 48, and 72 h, respectively. Thus, 227 μM (M0) and 48 h were selected as the intervention concentration and time of MPP^+^ in subsequent experiments. To determine whether the cytotoxicity of MPP^+^ against PC12 cells induces apoptosis, the present study analyzed nuclear morphological changes by AO/EB staining following 48 h of treatment with MPP^+^. As shown in Fig. 1b, the PC12 cells exhibited karyopyknosis and were notably stained with red following treatment with MPP^+^, the viable cells exhibited uniformly green. These results indicated that MPP^+^ could increase the apoptosis of PC12 cells in a dose-dependent manner.

### Glutamine suppressed cytotoxicity induced by MPP^+^ in PC12 cells

**Fig. 2:**
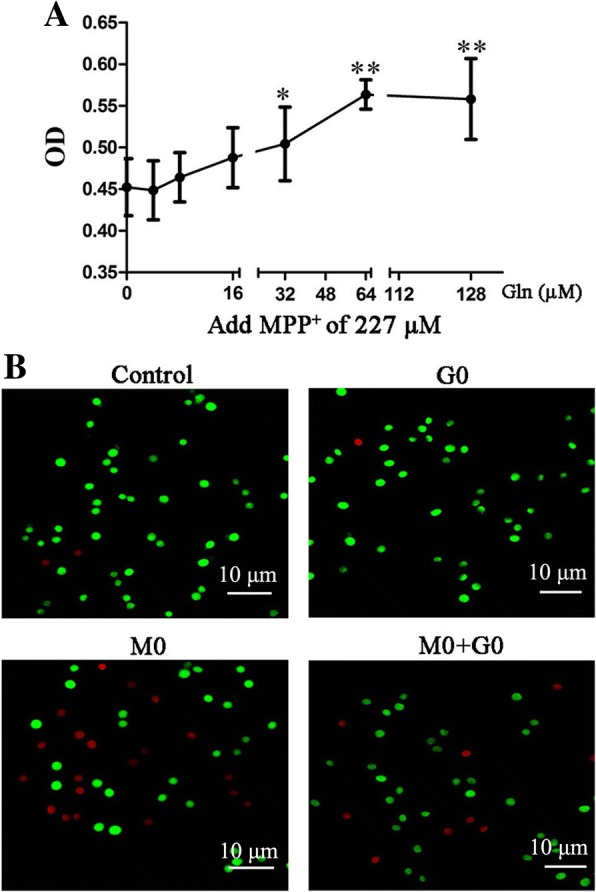
Protective effect of glutamine on MPP^+^-induced PC12 cell injury. **a** PC12 cells were incubated with MPP^+^ (227 μM) for 48 h following pre-treated with glutamine (0, 4, 8, 16, 32, 64, and 128 μM) for 1 h. Cell viability was determined by a Cell Counting Kit-8 assay. Data were obtained from three independent experiments. The results were presented as the mean ± standard deviation. *p < 0.05 and **p < 0.01, compared with Gln (0 μM). **b** PC12 cells were pre-treated with glutamine (64 μM) for 1 h, followed by incubation with or without MPP^+^ (227 μM) for 48 h. Images of AO/EB staining were observed via fluorescence microscopy (× 400), the 64 μM of glutamine is showed as G0

To evaluate whether glutamine has protective effect on MPP^+^-induced PC12 cell injury, cells were pre-treated with different concentrations of glutamine for 1 h, followed by incubation with MPP^+^ (227 μM) for 48 h. As shown in Fig. 2a, the viability of PC12 cells was significantly increased following pre-treatment with glutamine, and glutamine at 64 μM had the most significant protective effect on PC12 cells. Additionally, AO/EB staining demonstrated that glutamine combined with MPP^+^ could decrease cell apoptosis, indicating that glutamine plays a protective role against MPP^+^-induced PC12 cell apoptosis (Fig. 2b).

### Glutamine strengthens the antioxidant capacity in a PD cell model

**Fig. 3:**
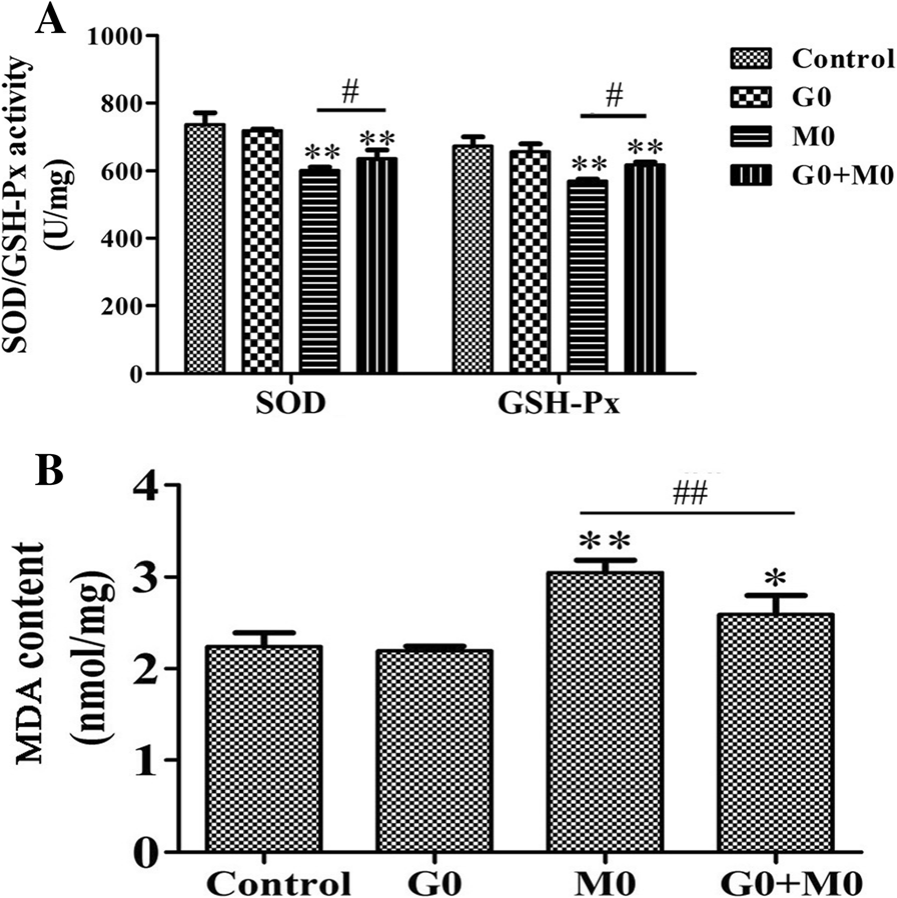
Effects of glutamine on SOD and GSH-Px activity and MDA content caused by MPP^+^ in PC12 cells. PC12 cells were incubated with MPP^+^ (227 μM) for 48 h following pre-treatment with glutamine (64 μM) for 1 h in G0+M0 group. **a** SOD and GSH-Px activity in each group. **b** MDA content in each group. Data were obtained from three independent experiments. The results were presented as the mean ± standard deviation. *p < 0.05 and **p < 0.01, compared with the control group; ^#^*p* < 0.05 and ^##^*p* < 0.01, compared with M0 group. SOD, superoxide dismutase; GSH-Px, glutathione peroxidase; MDA, malondialdehyde

As shown in Fig. 3, compared with the control group, the activity of SOD and GSH-Px was significantly decreased, while the content of MDA was markedly increased in the M0 group. Compared with the M0 group, the G0+M0 group displayed increased SOD and GSH-Px activity and reduced MDA content. These results indicated that glutamine could improve the antioxidant capacity of a PD cell model.

### Glutamine inhibits the activation of PI3K/Akt/mTOR signaling pathway in vitro

**Fig. 4:**
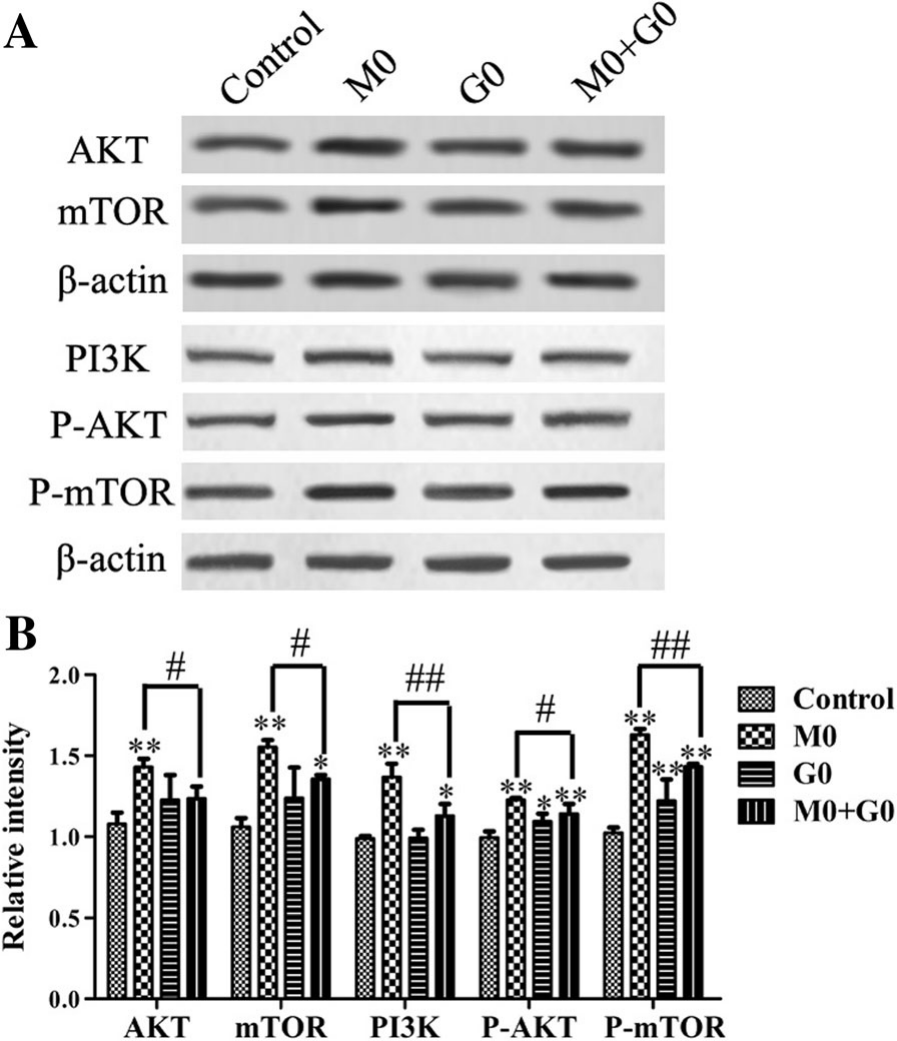
Protein expression levels of PI3K/Akt/mTOR pathway-related factors in the PD cell model after treatment with glutamine. **a** PC12 cells were incubated with MPP^+^ (227 μM) for 48 h following pre-treated with glutamine (64 μM) for 1 h, the key proteins of the PI3K/Akt/mTOR signaling pathway were examined by western blotting. **b** The relative intensity of proteins was shown as a bar graph. The experiments were repeated three times. The results were presented as the mean ± standard deviation. *p < 0.05 and **p < 0.01, vs the control group. ^#^*p* < 0.05 and ^##^*p* < 0.01, compared with M0 group

The western blot results indicated that the MPP^+^ could upregulate the expression levels of PI3K, P-Akt, Akt, P-mTOR, and mTOR, compared with the control group (Fig. 4). The expression levels of PI3K, P-Akt, Akt, P-mTOR, and mTOR in the M0+G0 group were significantly decreased compared with those in the M0 group, while markedly increased compared with the control group. These results suggested that glutamine could inhibit the activation of PI3K/Akt signaling pathway, but cannot reverse it.

### Glutamine strengthens the antioxidant capacity through inhibiting the PI3K/Akt/mTOR signaling pathway in vitro

**Fig. 5:**
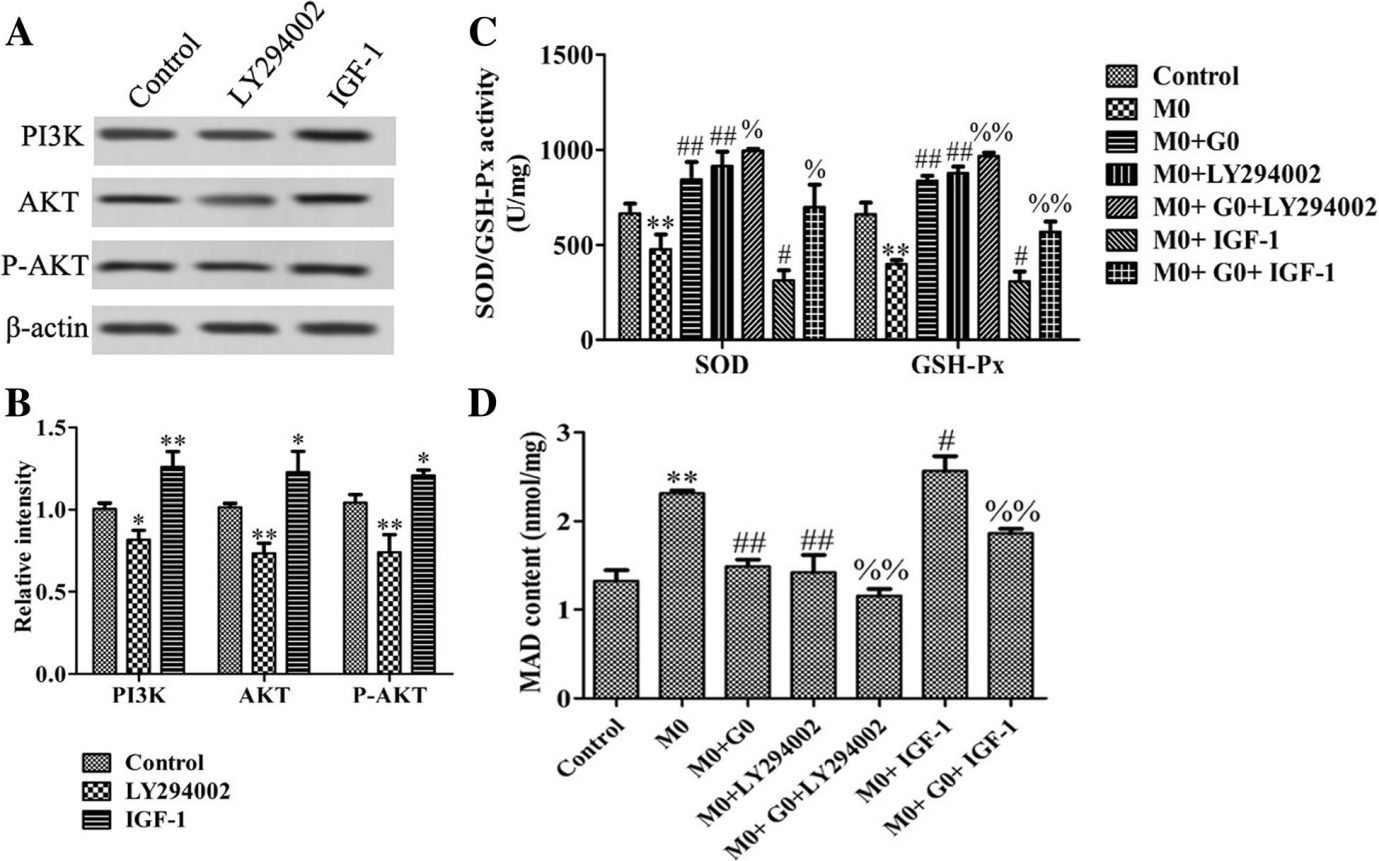
Effect of glutamine combined with LY294002 or IGF-1 on SOD and GSH-Px activity and MDA content caused by MPP^+^ in PC12 cells. **a** PC12 cells were incubated with LY294002 (20 μM) or IGF-1 (100 ng/ml). The PI3K, Akt, and P-AKT proteins were examined by western blotting. **b** The relative intensity of proteins was shown as a bar graph. **c** PC12 cells were incubated with MPP^+^ (227 μM) for 48 h following pre-treatment with glutamine (64 μM) and/or LY294002 (20 μM) and/or IGF-1 (100 ng/ml) for 1 h. SOD and GSH-Px contents in each group. **d** MDA content in each group. Data were obtained from three independent experiments. The results were presented as the mean ± standard deviation. *p < 0.05 and **p < 0.01, compared with control group. ^#^*p* < 0.05 and ^##^*p* < 0.01, compared with M0 group. ^%^*p* < 0.05 and ^%%^*p* < 0.01, compared with M0+G0 group. SOD, superoxide dismutase; GSH-Px, glutathione peroxidase; MDA, malondialdehyde

To elucidate whether PI3K/Akt/mTOR signaling pathway involved in glutamine protects PC12 cell against oxidative stress, LY294002 and IGF-1, PI3K/Akt signaling pathway inhibitor and agonist were used, respectively. As shown in Fig. 5a, b, the protein expression levels of PI3K, P-Akt, and Akt were significantly decreased in the LY294002 group and significantly increased in the IGF-1 group, compared with those in the control group, indicating that the PI3K/Akt/mTOR signaling pathway was successfully inhibited by LY294002. Compared with the control group, SOD and GSH-Px activity was significantly decreased, while MDA content was markedly increased in the M0 group. Compared with the M0 group, the activity of SOD and GSH-Px was significantly increased, while MDA content was markedly decreased in the M0+G0 and M0+LY294002 groups, indicating that both glutamine and LY294002 could decrease the oxidative stress level of PC12 cells. Compared with the M0 group, the SOD and GSH-Px activity was significantly decreased, while MDA content was markedly increased in the M0+IGF-1 group, indicating that the activation of PI3K/Akt/mTOR signaling pathway could increase the oxidative stress level of PC12 cells. Compared with the M0+G0 group, the SOD and GSH-Px activity was significantly increased in the M0+G0+LY294002 group and decreased in the M0+G0+IGF-1 group; MDA content was the opposite, indicating that the inhibition of the PI3K/Akt/mTOR pathway can enhance the antioxidant capacity of glutamine in the PD cell model (Fig. 5c, d).

## Discussion

MPP^+^ is an oxidative product of 1-methyl-4-phenyl-1, 2, 3, 6-tetrahydropyridine (MPTP), which can be transported into dopaminergic neurons and caused a severe parkinsonian syndrome [17, 18]. Thus, MPP^+^ is usually used to induce PD models in vivo and in vitro. Glutamine, as the most abundant free amino acid, has been proved to be closely related to the occurrence and development of neurodegenerative diseases [19]. Wang et al. reported that glutamine could inhibit alpha-Synuclein accumulation to protect cells against degeneration in the PD cell model [20]. In the present study, our results showed that the MPP^+^ displayed obvious cytotoxicity on PC12 cells, while glutamine could increase the viability of PC12 cells through suppressing neurotoxicity induced by MPP^+^. These results suggested that glutamine has a role in resisting neurotoxicity induced by MPP^+^.

It is widely accepted that oxidative stress plays a vital role in the degeneration of dopaminergic neurons in PD [21]. Previous studies demonstrated that glutamine had a positive role in preventing oxidative stress on human melanocyte and rabbit spermatozoa [22, 23]. Thus, the present study aimed to investigate the effects of glutamine on oxidative stress-induced injury in the PD cell model. SOD and GSH-Px are important antioxidant enzymes in organisms, which can remove superoxide free radicals and prevent the production of hydroxyl free radicals, respectively; MDA is a lipid peroxidation biomarker with cytotoxicity, which can indirectly reflect the degree of cell injury [24]. Our results showed that glutamine restored the decrease of SOD and GSH-Px activity and the increase of MDA content was induced by MPP^+^. Our findings revealed that glutamine could protect against oxidative stress-induced injury in the PD cell model.

Glutamine affects the process of various diseases by regulating oxidative stress [25, 26]. PI3K/Akt signaling cascade was the common final pathway for neuroprotection and self-repair through antioxidative stress [27]. Khallaghi et al. reported that metformin-induced protection against oxidative stress is associated with Akt/mTOR restoration in PC12 cells [28]. The inhibition of Rac1 could ameliorate neuronal oxidative stress damage through the PI3K/Akt/mTOR pathway [29]. Ruiqi et al. reported that the Luteolin ameliorates inorganic mercury-induced cardiac injury through the PI3K/Akt/Nrf2 signaling pathway to reduce oxidative stress levels [30]. Therefore, we speculated that glutamine could exert the effect of antioxidant stress through the PI3K/Akt signaling pathway, protecting cell survival. In the present study, our results showed that glutamine inhibited the activation of the PI3K/Akt signaling pathway in the PD cell model, indicating that the PI3K/Akt signaling pathway may play a negative regulatory role in glutamine suppressed cytotoxicity induced by MPP^+^. As expected, the inhibition of the PI3K/Akt signaling pathway can enhance the antioxidant capacity of glutamine in the PD cell model, while the activation of the PI3K/Akt signaling pathway relieved the antioxidant capacity of glutamine. These data suggested that glutamine strengthens the antioxidant capacity in PC12 cells through the PI3K/Akt signaling pathway.

## Conclusion

In summary, the current study indicates that glutamine plays a protective role in the PD cell model through reducing oxidative stress levels and inhibiting the activation of the PI3K/Akt signaling pathway. Furthermore, the block of PI3K/Akt axis could improve the antioxidant capacity of glutamine in the PD cell model. Taken together, we demonstrated that the mechanism of glutamine strengthens the antioxidant capacity in PC12 cells induced by MPP^+^ via inhibiting the activation of the PI3K/Akt signaling pathway, potentially offering new molecular targets for treatment of PD.

## Abbreviations

Akt: Protein kinase B
ANOVA: One-way analysis of variance
AO: Acridineorange
CCK-8: Cell Counting Kit-8
EB: Ethidium bromide
G0: 64 μM glutamine
Gln: Glutamine
GSH-Px: Glutathione peroxidase
M0: 227 μM MPP^+^
MDA: Malondialdehyde
MDS: Movement Disorder Society
MPP^+^: 1-Methyl-4-phenylpyridinium
MPTP: 1-Methyl-4-phenyl-1, 2, 3, 6-tetrahydropyridine
mTOR: Mammalian target of rapamycin
PD: Parkinson’s disease
PI3K: Phosphatidylinositol-3-kinase
PVDF: Polyvinylidene fluoride
SDS-PAGE: Sodium dodecyl sulfate-polyacrylamide gel electrophoresis
SOD: Superoxide dismutase
